# Keratoconus Following Cataract Extraction: A Patient-Centered Perspective

**DOI:** 10.7759/cureus.86635

**Published:** 2025-06-23

**Authors:** Firdhaus Zainudin, Yuen Keat Gan, Haireen Kamaruddin, Jemaima Che Hamzah

**Affiliations:** 1 Department of Ophthalmology, Hospital Selayang, Batu Caves, MYS; 2 Department of Ophthalmology, Universiti Kebangsaan Malaysia Medical Centre, Selangor, MYS; 3 Department of Ophthalmology, Universiti Kebangsaan Malaysia, Kuala Lumpur, MYS

**Keywords:** cataract patients, corneal astigmatism, irregular astigmatism, keratoconus (kc), phacoemulsification cataract surgery

## Abstract

Keratoconus is one of the most common forms of corneal ectasia. In severe cases, patients experience significant visual impairment, which can be further exacerbated by the development of cataracts. This deterioration may not only impact their vision but also affect their emotional well-being and mental health.

Here, we report a case of a patient with severe keratoconus who underwent cataract surgery in both eyes, leading to a remarkable transformation in her life.

An 80-year-old woman with underlying hypertension initially presented with bilateral blurring of vision and elevated intraocular pressure (IOP). She was diagnosed with bilateral primary angle closure glaucoma (PACG) and was started on antiglaucoma treatment, with cataract extractions planned.

Before surgery, she was semi-dependent and experienced frustration and depression due to her deteriorating vision. The preoperative assessment revealed keratoconus, with cylindrical power of -10.00DC in the right eye and -9.00DC in the left eye. She consented to monofocal toric intraocular lens (IOL) implantation.

Postoperatively, her cylindrical power was significantly reduced to -2.50DC in the right eye and -1.50DC in the left eye. Her vision improved markedly despite underlying PACG and tunnel vision. The patient now feels more independent and her quality of life has significantly improved.

Careful biometry calculations, thorough planning, and patient counseling are essential for achieving optimal postoperative outcomes in cataract surgery for stable keratoconus patients. This case highlights that significant astigmatism correction due to keratoconus is possible in selected individuals, improving both vision and overall quality of life.

## Introduction

Keratoconus is a noninflammatory corneal ectasia characterized by progressive thinning and protrusion, leading to a conical corneal shape and significant irregular astigmatism [1]. Astigmatism is a type of vision distortion caused by an uneven corneal surface, where two different axes of the cornea are parallel to each other. Irregular astigmatism is where the axes are not parallel to each other. Cataract surgery in keratoconus patients presents unique challenges, particularly in selecting the appropriate intraocular lens (IOL) and managing patient expectations to achieve optimal visual outcomes [2]. This case highlights the possibility of correcting severe astigmatism secondary to keratoconus in selected individuals, demonstrating the potential for improved vision and overall quality of life.

Verbal and written consent was obtained from the patient for the publication of the article for educational purposes.

## Case presentation

An 80-year-old woman with a history of hypertension presented with a gradual onset of blurred vision in the left eye, persisting for one year. She reported no other ocular symptoms such as pain, floaters, or visual field defects.

On examination, her visual acuity (VA) was recorded as 6/45 in the right eye and counting fingers (CF) in the left. A grade 2 positive relative pupillary afferent defect (RAPD) was observed in the left eye. Intraocular pressure (IOP) measurements were 19 mmHg for the right eye and 24 mmHg for the left. Gonioscopy revealed closed angles in both eyes, and both lenses were cataractous. Fundus examination showed a cup-to-disc ratio (CDR) of 0.9 with glaucomatous optic neuropathy changes bilaterally.

**Figure 1:**
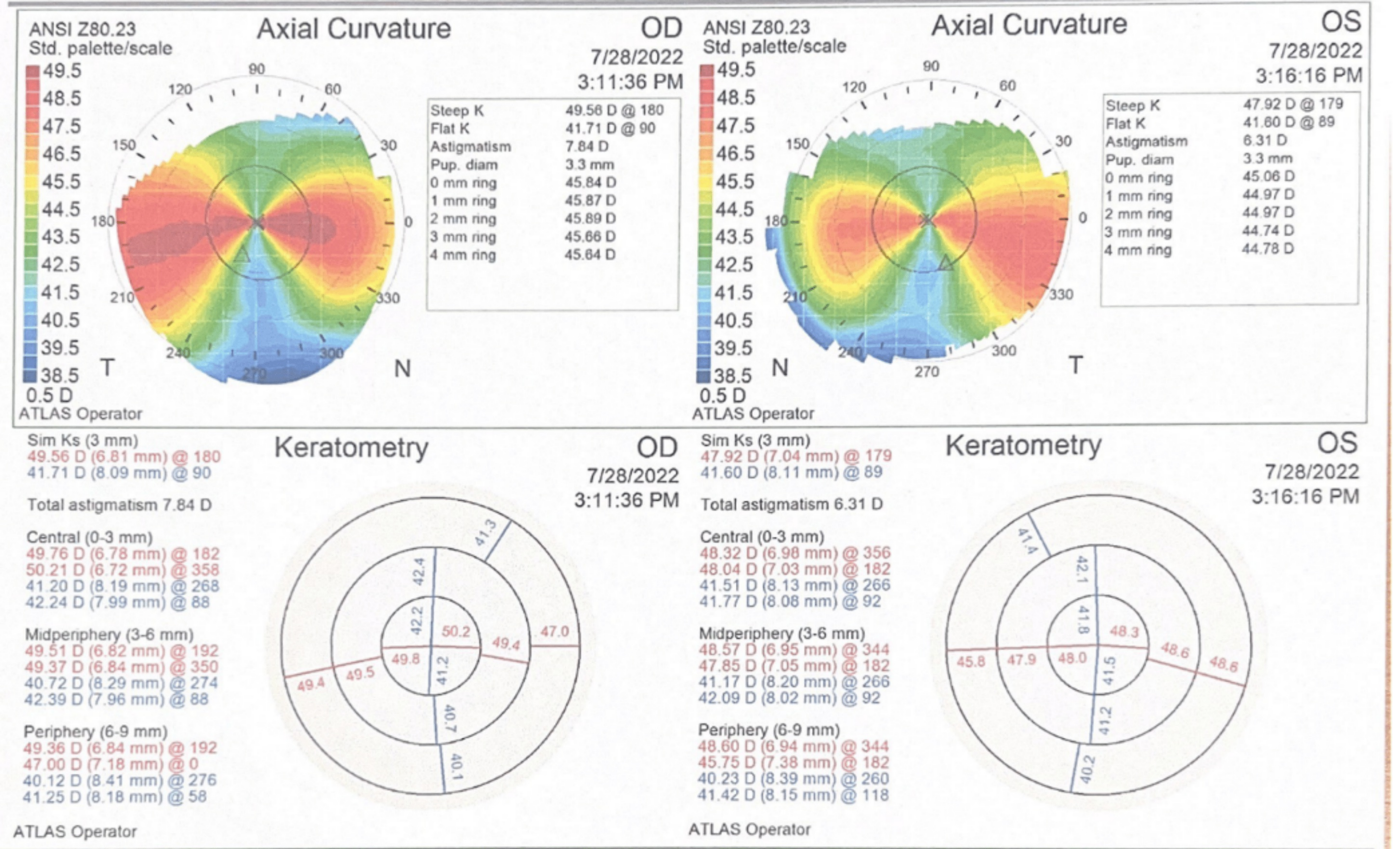
The topography reveals an irregular bowtie pattern with elevated keratometry readings, indicating significant corneal curvature irregularities. However, no evidence of corneal thinning is observed.

She was diagnosed with bilateral primary angle closure glaucoma (PACG), started on antiglaucoma therapy, and scheduled for cataract extraction. The preoperative assessment revealed a high cylindrical refractive error, measuring -10.00 DC in the right eye and -9.00 DC in the left. Her corneal topography findings are presented in Figure 1. Other ocular biometry parameters remained within normal limits.

Further questioning revealed that family members had observed the patient becoming increasingly semi-dependent, expressing frustration and depression due to her visual impairment. She had previously worn glasses but struggled with intolerance due to high astigmatism.

The patient was counseled and offered cataract extraction with monofocal toric IOL implantation. We aimed for a slight low myopic shift for both eyes, and the lens was implanted along the steep axis. She underwent bilateral cataract surgery in separate sessions, both of which were uneventful. Postoperative follow-ups showed well-controlled IOP and no progression of glaucoma or keratoconus.

Final refraction demonstrated a significant reduction in cylindrical power to -2.50 DC in the right eye and -1.50 DC in the left. Her best corrected visual acuity (BCVA) was 6/9.5 for the right eye and 6/38 for the left eye. The patient was delighted with her improved vision despite underlying PACG and tunnel vision in the left eye.

Following surgery, she reported feeling more confident and independent. According to her, her mood and emotional well-being improved, leading to an overall enhancement in her quality of life. As her keratoconus remained stable, no further intervention was required.

## Discussion

The management of keratoconus requires a holistic approach tailored to fulfill patient needs. Visual intervention primarily consists of spectacles or contact lenses, such as rigid gas permeable (RGP) lenses [1]. In more advanced cases, surgical options include corneal cross-linking, intracorneal ring segments (ICRS), deep anterior lamellar keratoplasty (DALK), and penetrating keratoplasty (PK) for severe cases with corneal opacity [1].

In cataract surgery for keratoconus patients, the key challenge lies in achieving the BCVA. A meticulous preoperative assessment is crucial to ensure accurate and reproducible keratometry and axial length (AXL) measurements [2]. Additionally, careful preoperative planning is necessary, particularly in selecting the most appropriate IOL formula [2,3]. Standard correction assumptions for corneal curvature are not uniform across different keratometric values in keratoconic eyes [2].

The SRK-T and SRK II formulas have been found to be suitable for mild to moderate keratoconus patients [3]. However, newer formulas such as Holliday II, Barret Universal II, and other 4th-generation formulas continue to emerge, offering more reliable and precise calculations tailored to the evolving nature of keratoconus. For our patient, we used the Barrett True-K formula, which requires both anterior and posterior corneal keratometry readings. While studies suggest that most calculations result in a hyperopic shift [3], our patient experienced a myopic shift in refraction, which can be influenced by the surgeon's experience and preference. Therefore, no accurate study has been proven for keratoconus [3].

Monofocal toric IOLs are recommended for mild to moderate keratoconus [4]. In severe cases, toric IOLs may be less suitable due to the difficulty in predicting postoperative outcomes [4]. IOL selection ultimately depends on surgeon comfort and availability, which varies across medical centers.

Thorough preoperative counseling is essential to understand patient expectations and discuss potential outcomes. Patient selection and meticulous surgical planning help minimize complications. During surgery, any appropriate method can be employed, but complete removal of the lens nucleus and cortex, along with careful ophthalmic viscosurgical device (OVD) removal, is necessary to prevent postoperative IOL rotation. Comprehensive postoperative care is equally important in reducing the risk of sight-threatening complications such as endophthalmitis.

Morshifar et al. reported that some patients may require rigid RGP lenses after cataract surgery if there is evidence of keratoconus progression or to prevent further deterioration [2]. Additionally, ICRS implantation has been shown to improve visual outcomes following cataract extraction [3]. Combining cataract extraction and IOL implantation with ICRS implantation may help minimize the need for multiple surgeries [4,5].

## Conclusions

Keratoconus management continues to evolve with advancements in diagnostic tools, surgical techniques, and IOL formulas. Ophthalmologists must stay informed about both traditional and emerging approaches to optimize patient care. Well-planned preoperative and intraoperative management strategies help reduce complications and enhance visual outcomes in cataract surgery for keratoconus patients.
